# Spiders Once More

**Published:** 1888-05

**Authors:** 


### SPIDERS ONCE MORE.

We translate the following from the Parisian *Journal D' Hygiene*, which gives some additional facts respecting spiders, called out by our former article.

Animals, even the humblest of them, have their peculiar characteristics portrayed in the written legends which perpetuate them from age to age.

It is owing to these traditions that the spiders, these modest and useful rural laborers, have been at all times and in divers countries, the object of singular superstitions.

We learn from HALL'S JOURNAL OF HEALTH, that in England spiders are piously respected and guarded against disturbance, lest some great misfortune overtake one.

> " If good fortune you would win
> Let the spider live and spin,"

says a proverb on the other side of the channel.

This veneration is sufficiently illustrated by a popular story of a a spider who had woven his web above the manger at Bethlehem, where the infant Jesus escaped dangers of all sorts with which he was menaced. It was also a spider which saved the life of King David in the desert of Ziph, by weaving before him a web, under cover of which he was able to make his escape, safe and sound, by a dangerous passage. A like respect of these spider-webs is discovered in oriental traditions. Mahomet, in the course of his flight and concealment from Mecca owed his safety to a spider and a pigeon. The first had woven its web before the entrance to the cave in which the prophet had taken refuge, and the pigeon had built her nest above it, so that the soldiers who pursued him, doubting not for a moment that the cave had been deserted for a long time, passed it by without examination.

It is a Scotch proverb that "he who kills a spider in the morning will, of necessity, break something during the day." In this country

(France) the discovery in a room of a "money-spinner, a particular species of spider, is a certain indication of prosperity, which sign, accord- to a popular adage, will never deceive one. * * *

There is a story to the effect that Robert Bruce, the old-time Scotch patriot, after several fruitless attempts to liberate his country, was lying awake upon his cot, early one morning, when his attention was drawn to a spider overhead in an attempt to attach its web at a difficult point. After repeated failures, corresponding in number to those of Bruce, it succeeded. The hero accepted the omen, which did not deceive him. Thus it is seen that a harmless superstition may sometimes serve a good purpose.

